# Effort in Multitasking: Local and Global Assessment of Effort

**DOI:** 10.3389/fpsyg.2017.00111

**Published:** 2017-02-06

**Authors:** Andrea Kiesel, David Dignath

**Affiliations:** Department of Psychology, Cognition, Action, and Sustainability Unit, University of FreiburgFreiburg, Germany

**Keywords:** multitasking, task switching, cognitive control, voluntary action, voluntary task switching

## Abstract

When performing multiple tasks in succession, self-organization of task order might be superior compared to external-controlled task schedules, because self-organization allows optimizing processing modes and thus reduces switch costs, and it increases commitment to task goals. However, self-organization is an additional executive control process that is not required if task order is externally specified and as such it is considered as time-consuming and effortful. To compare self-organized and externally controlled task scheduling, we suggest assessing global subjective and objectives measures of effort in addition to local performance measures. In our new experimental approach, we combined characteristics of dual tasking settings and task switching settings and compared local and global measures of effort in a condition with free choice of task sequence and a condition with cued task sequence. In a multi-tasking environment, participants chose the task order while the task requirement of the not-yet-performed task remained the same. This task preview allowed participants to work on the previously non-chosen items in parallel and resulted in faster responses and fewer errors in task switch trials than in task repetition trials. The free-choice group profited more from this task preview than the cued group when considering local performance measures. Nevertheless, the free-choice group invested more effort than the cued group when considering global measures. Thus, self-organization in task scheduling seems to be effortful even in conditions in which it is beneficiary for task processing. In a second experiment, we reduced the possibility of task preview for the not-yet-performed tasks in order to hinder efficient self-organization. Here neither local nor global measures revealed substantial differences between the free-choice and a cued task sequence condition. Based on the results of both experiments, we suggest that global assessment of effort in addition to local performance measures might be a useful tool for multitasking research.

## Introduction

In everyday life multiple cognitive task requirements are omnipresent and occur in many different contexts. For example, teachers concurrently observe the behavior of problematic pupils while they are engaged in explaining a mathematical procedure, a text passage, etc. Surgeons have to concurrently track the vital functions of the patient while they are engaged in opening the ribcage. Working in an office requires performing cognitive tasks like planning the budget or evaluating the outcome of the work group, and these tasks might be interrupted by phone calls, incoming emails or colleagues/students knocking at the door. And, finally, managing a household with children permanently requires engaging and disengaging in several tasks like planning a dinner, looking out for sources of dangers for just-walking children, answering questions of older children etc. Thus, multiple cognitive task requirements are a societal fact and one can hardly avoid them.

While multitasking is generally costly, there might be factors that help people to cope better with multitasking. Self-organization of task choice and task scheduling certainly is such a factor. However, self-organization is also a process that requires additional control. Consequently, we reason that while some conditions might be beneficial for multitasking in terms of better task performance, this will come at a cost in terms of more effort required. Therefore, we focus not only on local performance measures, but also on global measures of effort in terms of subjective and objective measures. As a first step, we aim to present a new experimental approach to compare conditions in which participants themselves organize how to cope with multiple cognitive task requirements with conditions in which task organization is externally controlled and thus task scheduling is pre-determined. In the experimental task, we combine properties of dual tasking and task switching paradigms by allowing for parallel processing of different tasks in a protocol that requires the rapid alternation between tasks. By this, we present an experimental set-up that allows the independent assessment of local and global costs of self-organization processes during multitasking. While the process of self-organization itself has been investigated elsewhere, the focus of this research is on a possible trade-off between local and global measures.

The comparison between self-organized and externally controlled task scheduling is empirically and theoretically especially interesting because different lines of psychological research allow opposing hypotheses. First, research in PRP studies that allowed participants to freely choose task order, revealed that several factors, like for example, expectation of stimulus order or repetition of task order (De Jong, 1995), distribution of stimulus onset asynchronies between Task 1 and Task 2 stimuli (Miller et al., 2009), duration of central processing stages of Task 1 and 2 (Leonhard et al., 2011; Ruiz Fernández et al., 2011) or duration of motor responses (Ruiz Fernández et al., 2013) impact on whether participants perform Task 1 or Task 2 first. These findings are in line with the assumption of a higher-order control process that determines task order and preparation for the tasks (e.g., De Jong, 1995; Luria and Meiran, 2003; Szameitat et al., 2006). Recent theorizing assumed that task order in conditions with varying distribution of stimulus onset asynchronies might be chosen in a way that optimizes task performance (see Miller et al., 2009 for an optimization account). Consequently, conditions that enable to self-organize task order might be advantageous compared to conditions with externally controlled task order.

Similarly, recent research in the voluntary task switching paradigm suggests that self-organization might be advantageous over cued task switching. In voluntary task switching settings, participants freely choose which task to perform in the next trial whereas in cued task switching settings, a cue is presented prior to each target instructing participants which task to perform in the next trial. Switch costs, that is the RT difference for task switch and task repetition trials, are smaller in voluntary task switching settings compared to cued task switching (e.g., Arrington and Logan, 2005; Mayr and Bell, 2006; Demanet and Liefooghe, 2014).

And finally, within applied work psychology, researchers predict that self-organized task performance is superior to fixed task scheduling. With regards to this assumption, the self-regulation theory of Hacker (e.g., Hacker, 2009) claims that goals and plans are relevant to regulate one’s action. Further, commitment to these goals seems especially high if workers participate in the goal-setting process (e.g., Pritchard et al., 1993; Kleinbeck and Schmidt, 2004). Indeed, there are even norms that request holistic and complete work activities (ISO 6385, EN DIN 29241-2, cited in Hacker, 2009). Thus, from this perspective self-organized task-scheduling likewise might be considered as favorable compared to fixed task scheduling.

On the other hand, however, self-organization is an extra cognitive processes that is not required if task order is externally controlled. This process to choose tasks/task order to optimize performance is conceptualized as an executive control process (e.g., Logan, 1985; Norman and Shallice, 1986) and as such it is considered as time-consuming and effortful. However, previous research might not be ideal to investigate this process for several reasons. For instance, task choice often takes place prior to stimulus presentation. We argue that if this is the case, participants cannot actually choose task order to optimize their performance because they do not know the exact task requirements for the respective trial. Instead participants have to base their task choice on rather broad requirements of the tasks in general. Consider the case of a participant choosing between a math and letter identification task. If the participant has to decide prior to stimulus presentation, she can recall some rather abstract features of the task (e.g., in the math task, I have to do simple computations) and will probably base her decision on her assessment of the *anticipated* level of difficulty. We will call this a proactive, memory-driven strategy. In contrast, if the participant has to decide after stimulus presentation, she can compare the different items based on specific features (e.g., in the math task, I have to subtract 4 from 9). Consequently, her decision will be based on her assessment of the *actual* level of difficulty. We will refer to this as a reactive, stimulus-driven strategy. Arguably, it is much easier to choose the’ best’ task (in terms of time and effort invested in solving the task) if participants can apply a stimulus-driven strategy, because the memory-driven strategy has two disadvantages. First, with a memory-driven strategy, optimization is restricted to abstract features and therefore preparation will be necessarily limited. Second, with limited time and more than two alternatives, memory recall will be rather demanding, making it less likely those participants will use this strategy at all.

Further, we aim to assess effort and performance in a multitasking setting that actually requires participants to schedule task order. For this, we opted for a setting in which the items of the non-chosen tasks remained the same. Thus, participants are not simply able to apply a stimulus-driven strategy to choose the item that is easiest to perform in a given trial, but they are able to choose task order such that the order of items is optimized (at least to some degree for the respectively next items of each task). By this, our setting also better resembles everyday multitasking because we require participants to schedule the task order while the affordances (e.g., stimuli for a task) for the not-yet-performed tasks remain^1^. To summarize, while previous research investigated task choice mostly for memory-driven strategies, we believe that this actually limited the possibilities to optimize the task choice process. Therefore, the present research aimed to maximize the possibility that participants make use of a stimulus-driven strategy. More precisely, we reason that presentation of specific items prior to task choice will most likely facilitate performance, because participants can select (and solve) tasks based on their actual difficulty.

However, if task choice is optimized (in terms of better performance), the cognitive effort related to this optimization process cannot be assessed with traditional measures of task performance. Indeed, research in PRP and task switching settings usually did not assess overall effort to handle the experimental requirements. Consequently, to arrive at a more complete picture of task optimization, it is crucial to assess both global and local measures when comparing effort for self-organized compared to externally controlled task scheduling.

In this paper, we aim to consider both – local performance measures and global effort measure to compare self-organized and externally controlled task scheduling when confronted with multiple cognitive requirements. Indeed, there are many studies comparing voluntary and cued task switching performance while assessing local performance data. Yet, results are ambiguous. Although studies unequivocally revealed that switch costs are smaller in voluntary task switching settings compared to cued task switching (e.g., Arrington and Logan, 2005; Mayr and Bell, 2006; Demanet and Liefooghe, 2014), the result patterns diverge when considering overall RT level. Mayr and Bell (2006) and Demanet and Liefooghe (2014) observed faster RTs for voluntary task choice compared to cued task order, yet Arrington and Logan (2005) reported in 4 of 5 experiments slower RTs for voluntary task choice compared to cued task order (see also Chen and Hsieh, 2013).

In addition, studies comparing performance in voluntary and cued task switching settings usually did not control for task transition effects. In cued task switching settings, task order is random and consequently frequency of task switches is approximately 50% (for settings with two tasks). Yet, if participants freely choose tasks, frequency of task switches usually differs from chance because participants repeat tasks more often as expected by random task choices (e.g., Arrington and Logan, 2004, 2005; Mayr and Bell, 2006; Yeung, 2010; Reuss et al., 2011). A fair comparison of performance in voluntary and cued task switching settings requires controlling for task transition effects. This is intended in the current study by applying a yoked design. That is, for each participant in the free choice condition, there is one participant in the cued condition who is cued to perform the tasks in exactly the same task order as chosen by the participant in the free choice condition (for a similar attempt see Panepinto, 2010; Masson and Carruthers, 2014).

To conclude, experimental settings in voluntary task switching studies differ from everyday task performance and do no foster task scheduling that optimizes performance. Participants have to choose which type of task to perform without knowing the exact task requirements based on a memory-driven strategy. In addition, the items for the non-chosen task do not remain while in everyday life not-yet-performed task requirements usually do not change. Indeed, only if the exact task item is known, participants can choose a task and/ or select task order according to a stimulus driven strategy, allowing for an optimization of task choices.

## Experiment 1

To compare performance and effort for self-organized compared to externally controlled multiple cognitive task requirements, we compared a free-choice group and a cued task switching group in a yoked design. We applied a new experimental paradigm that combines characteristics of PRP and task switching settings. Participants were requested to perform four different tasks: a summation task, a subtraction task, a distance month task, and an alphabetical distance task. For the summation task and the subtraction task, two one-digit numbers had to be added or subtracted. The distance month task required counting the amount of months from a start to an end month. For example, the item “January > > February” required the response 1 and the item “July > > January” required the response 6. The alphabetical distance task required counting the amount of letters from a start to an end letter. For example, the item “H > > L” required the response 4. The respective items for the four tasks were chosen such that all items are responded to with one-digit numbers; participants pressed the corresponding numbers of the number pad of a standard keyboard.

**FIGURE 1:**
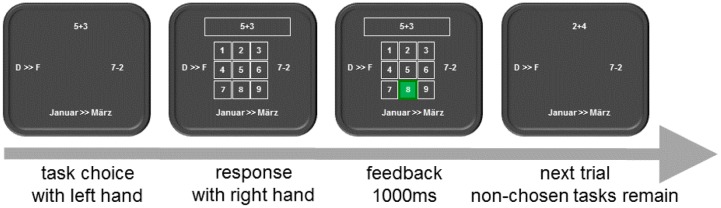
**Trial sequence.** Participants in the free choice condition choose a task with their left hand and type in the correct digit with their right hand. The trials sequence for participants in the cued condition was similar except that participants simply started the trial by pressing a key.

Each task was presented at a fixed location on the screen (location and task mapping was counterbalanced between participants). Most importantly, participants simultaneously saw one item for each task (see **Figure 1**) and each specific item remained on the screen as long as the participant did not answer to this item. Thus, the respectively next items for the four tasks were presented in parallel and consequently participants could operate on the tasks simultaneously (like in PRP studies). Yet, responding to each task was strictly sequential (like in task switching paradigms). In each trial, the actual relevant task first had to be determined.

In the “free choice condition,” participants themselves indicated which task they chose by a left-hand response. Then a rectangle appeared that surrounded the item of the chosen task to confirm this task choice and participants responded to the item of this task. After responding, feedback was shown for 1000 ms before the next trial started. A new item was presented at the location of the performed task; the items that had not been responded to remained on the screen. Thus, during the feedback screen participants could use the preview of the items for the non-chosen tasks. We instructed participants in the free choice condition to choose tasks to respond as fast and as accurate as possible without following a predetermined strategy like for example to choose tasks in clock-wise order or to always alternate between two tasks.

To equalize the number of responses of the free choice and cued task condition, participants in the cued task condition were requested to press any of the four task keys to start the next trial. Then a rectangle appeared surrounding the item of one task to indicate that this was the currently relevant task. Similar to the free choice condition, participants in the cued group were encouraged to respond as fast and as accurate as possible. However, unlike to the free choice condition, participants did not know which item would be required in the next trial. Consequently, participants in the free choice condition could use the preview to work on an item that they would choose while participants in the cued condition could use the preview to work on any of the three remaining items yet without knowing when this item will be required. Please note, however, that even in the forced choice condition, it is perfectly rational to use the preview to work on any of the three remaining items because each single item remained on the screen until it became cued/relevant.

In order to compare free choice and cued task conditions, we considered several *global* measures to analyze whether conditions were differentially stressful/effortful. First, the concept of ego-depletion (Baumeister et al., 1998) assumes that self-control and choice processes are resource-consuming and lead to fatigue, that is impairment in a subsequently required unrelated task. In order to assess fatigue, participants performed a Stroop task that followed the main experiment (e.g., Webb and Sheeran, 2003; Inzlicht et al., 2006; Gailliot et al., 2007). Further, we assessed the amount of subjectively experienced stress on a scale by Eilers et al. (1986), and the amount of payment participants would consider fair for this kind of work (Thaler, 1980). To control for changes in mood, we assessed affect with explicit rating and with the “implicite positive and negative affect test” (IPANAT, Quirin et al., 2009).

In addition, we considered *local* task performance measures (RT and error rates) depending on whether participants switched or repeated the task, and we considered time to start the trial (time to choose a task in the free choice condition, and time to start the trial in the cued condition). To get a combined measure, we additionally computed the total work time, that is the sum of RT and time to start the trial. Please note that with this design, RT is measured from the onset of the task choice response (free choice condition) or the onset of the response to start the trial (cued task condition) until response. Because the items for each task were presented on the screen before, RT does not indicate the core time to perform this item. Thus, it is necessary to conjointly consider the time until participants choose a task/started the trial and the RT to assess the total work time. Further, we assessed the characteristic of the task choices in free choice condition. In addition to frequency of repetitions, we assessed whether switches occurred between task categories or across task categories. Within this regard, we considered the summation and subtraction task as one task category and the distance month task and the alphabetical distance task as another task category because of the similarities regarding stimuli (numbers vs. words/letters) and required cognitive operation (computation vs. distance assessment).

### Method

#### Participants

Forty-eight participants were paid 10 for participation. Data of one participant had to be excluded due to technical problems and data of another participant were excluded because the participant did not finish the experiment in the given time slot. To control for task transitions, we also excluded the data of the respectively yoked participants. Thus, data of 44 participants (seven men, two left-handed, 18–56 years) were analyzed. All participants were tested within 2 weeks in sessions that lasted approximately 90 min. The first 10 participants were assigned to the voluntary group and the next ten participants were yoked to the first 10 participants and tested under the cue condition. This procedure was repeated for the remaining participants.

#### Stimuli

In the main experimental task, target stimuli were presented in white (Courrier New, 18) on black background. Tasks comprised of two simple math tasks that required the addition or subtraction of two digits (results ranged from 1 to 9, e.g., “3-2”) and of two simple counting tasks. In the counting tasks, participants were to indicate either the numerical distance between two letters (with a maximal distance of 6, e.g., “G > > L”) or between two calendar months (likewise with a maximal distance of 6, e.g., March > > January). Each task comprised of 36 different items.

In the Stroop task, color words were the German words for “BLUE,” “GREEN,” “YELLOW” and “RED” printed in blue, green, yellow, or red. For congruent words, the print color of the word matched the meaning of the word, while for incongruent words both colors mismatched. Only four combinations of incongruent words were presented to a specific participant to ensure presentation of individual congruent and incongruent Stroop items in equal frequencies (c.f. Melara and Algom, 2003). For this subset of incongruent color words, the assignment of the ink color to the meaning of the color word was counterbalanced across participants.

#### Procedure

##### Main experimental (task switching) task

Participants performed nine blocks and in each block participants had to respond to 36 items per task, thus in total to 144 items per block. The first block was considered as training block and was not analyzed. Each task was presented at a fixed location on the screen (counterbalanced between participants). The four different task items were presented around a central fixation cross. Importantly, participants simultaneously saw one item for each task (see **Figure 1**). Thus, the respectively next items for the four tasks were presented in parallel and consequently participants could operate on the tasks simultaneously.

In the free choice group, participants indicated their task choice with an overt response (cf. Arrington and Logan, 2005) by pressing the keys “w,” “a,” “s,” or “d” using the index finger of their left hand. Participants answered the tasks using the index finger of their right hand by pressing the numbers 1 – 9 on the number block. Participants were instructed to perform the tasks as fast as possible. Regarding task choice they were asked to choose in each trial the items they wanted to without following a fixed pre-determined strategy like for example rotating task-order clockwise and without choosing the same task more than 2 or 3 times in a row.

**Figure 1** shows the sequence of events in an experimental trial for the free choice group. A fixation cross was presented on the middle of the screen surrounded by the four tasks until a task was selected with a spatially congruent key press. The selected task was marked with a white frame and the fixation cross was replaced by a matrix of digits from 1 to 9. When a response was registered, the background color of the corresponding digit changed for 1000 ms from black to green in case of correct response or to red in case of an error. The next trial started directly with the presentation of a new item at the location of the just performed task; the non-chosen items remained on the screen. Items of a task were randomly administered. Whenever a participant had performed all 36 items of a task in a block, the signs “XXXX” were presented at the task location and participants had to choose among the remaining tasks. After each block there was a break and participants received feedback about the number of errors and the total time it took them to perform all tasks in the last block. When participants felt ready for the next block they terminated the break.

The procedure for participants in the cued group was identical, except that participants did not select task by themselves, but started a ‘random generator’ by pressing a start key (the same keys served as start keys than in the free choice group). The presented task and trial sequences, however, were not random, but yoked to one of the participants in the free choice group.

##### Stroop task

After performing the task switching experiment, participants were instructed to respond to the ink color of a word by pressing the keys ‘a,’ ‘x,’ ‘l’ or ‘m’ using the index and middle fingers of their left and right hands. The assignment of the response buttons to the ink color was counterbalanced across participants. At the start of a trial, a fixation-cross was presented for 300 ms followed by a colored word which prompted the participant to respond as quickly as possible. After 1000 ms, a blank screen was presented until registration of a key press. In case of an incorrect or late response (RT > 1000 ms), an error message appeared for 1000 ms. The next trial started after an intertrial interval of 1000 ms. The Stroop task consisted of four blocks with eight congruent and eight incongruent trials each.

##### Questionnaires

To assess explicit affect rating, participants indicated their current mood by clicking with the mouse cursor on a scale from 0 [very negative] – 100 [very positive] directly before and after the main experimental task (i.e., the task switching part). After performing the Stroop task, participants filled out the “implicit positive and negative affect test” (IPANAT, Quirin et al., 2009).

To assess subjective experience of fatigue and demand, participants answered the “scale to assess subjective experience of stress” (Eilers et al., 1986)^2^. Furthermore, we adopted a “compensation demanded measure,” a standard procedure from behavioral economics (e.g., Thaler, 1980; Knetsch and Sinden, 1984) to assess how much payment per hour participants considered as a fair compensation for their participation in the experiment.

### Results

#### Global Measures to Assess Fatigue/Stress

##### Stroop task

After the experiment, participants performed a Stroop task to assess fatigue. For the analysis, the first trial of the block was excluded and for the RT analyses, only correct trials were included. Participants responded slower, *F*(1,42) = 11.86, *p* = 0.001, $\eta_{p}^{2}$ = 0.220 and made more errors, *F*(1,42) = 21.29, *p* < 0.001, $\eta_{p}^{2}$ = 0.336 in Stroop incongruent compared to Stroop congruent trials^3^. Most importantly, participants in the free choice condition committed overall more errors than participants in the cued condition, *F*(1,42) = 4.99, *p* = 0.031, $\eta_{p}^{2}$ = 0.106, while response time did not differ significantly but also did not indicate any speed-accuracy tradeoff, *F*(1,42) = 2.60, *p* = 0.114, $\eta_{p}^{2}$ = 0.058, see **Table 1** for means.

**Table 1:** Mean RTs, error rates, and mean values (standard error in parenthesis) for the Stroop performance and the administered questionnaires.

	Stroop task						Subjective ratings					
	Congruent RT (ms)	Incongruent RT (ms)	Mean RT (ms)	Congruent error (%)	Incongruent error (%)	Mean error (%)	Effort	Compensation demanded ()	Affect explicit	Affect implicit positive	Affect implicit negative
Free-choice 1000 ms	578 (14)	601 (16)	589 (17)	39.0 (4.6)	51.5 (5.5)	45.1 (4.8)	115.7 (6.9)	25.2 (6.0)	51.8 (5.1)	1.9 (0.7)	1.7 (0.8)
Cued 1000 ms	536 (14)	566 (16)	551 (17)	25.4 (4.6)	34.2 (5.5)	29.8 (4.8)	92.4 (8.6)	12.5 (0.68)	46.9 (4.3)	1.8 (1.1)	1.6 (1.0)
Free-choice 200 ms	601 (14)	635 (15)	618 (10)	16.6 (3.8)	29.1 (4.2)	22.9 (3.7)	113.5 (8.3)	12.0 (3.7)	66.6 (4.2)	–	–
Cued 200 ms	599 (14)	639 (15)	619 (10)	22.4 (3.8)	30.4 (4.2)	26.4 (3.7)	96.4 (8.3)	11.6 (4.3)	61.7 (3.6)	–	–


##### Subjectively experienced stress

Participants reported more subjectively experienced stress in the free choice condition compared to the cued condition, *t*(42) = 2.1, *p* = 0.041. The amount of payment per hours that participants demanded for compensation for a future participation differed between conditions, *t*(39^4^) = 2.2, *p* = 0.036. Participants in the free choice condition indicated that 25,15 Euro per hour would be a fair payment for this work while participants in the cued task group considered 12,48 Euro per hour as fair payment.

##### Affect

In order to test the influence of choice condition on explicit affect, while controlling for potential differences in pre-test affect, we used the analysis of covariance approach (Senn, 2006). Post-test affect rating were entered into a univariate ANCOVA with choice condition (free vs. cued) as the between-participants factor and pre-test mean Mood ratings as the covariate. This analysis revealed no significant difference between groups, *F* < 1.

Implicit affect rating assessed by the IPANAT did not differ between groups, neither for positive affect nor negative affect, both |t| < 1.^5^

Taken together, participants in the free choice condition were more fatigued and experienced more stress than participants in the cued condition, yet this was not due to any impact on affect but seems to indicate that this condition is more effortful.

#### Local Task Switching Performance

The first trial in each block was not analyzed. Post-error trials (6.3%) and RTs that exceeded more than 2.5 SDs from the cell mean for each condition (4.4%) were removed from all analyses. Additionally trials with erroneous responses (5.2%) were removed from all analyses (except analysis of error data). If not stated otherwise, a repeated-measures analysis of variance (ANOVA) with the factors *condition* (free choice, cued) and *task transition* (repeat, switch) was used to analyze the data.

##### Task performance (reaction times and errors)

Participants in the free choice condition responded faster than participants in the cued condition, *F*(1,42) = 22.6, *p* < 0.001, $\eta_{p}^{2}$ = 0.35. Participants responded faster in task switch than in task repetition trials, *F*(1,42) = 17.7, *p* < 0.001, $\eta_{p}^{2}$ = 0.30. In addition, participants made less errors in task switch than in task repetition trials, *F*(1,42) = 4.8, *p* = 0.03, $\eta_{p}^{2}$ = 0.10, and this switch advantage in errors occurred mainly in the free choice group, *F*(1,42) = 4.1, *p* = 0.05, $\eta_{p}^{2}$ = 0.09. All other effects were not significant (*p* > 0.45).

##### Task choice times

Regarding the time to choose a task/start the trial, participants in the free choice condition took longer than participants in the cued task condition, *F*(1,42) = 14.5, *p* < 0.001, $\eta_{p}^{2}$ = 0.26, and especially so when they repeated tasks rather than switched tasks, *F*(1,42) = 26.7, *p* < 0.001, $\eta_{p}^{2}$ = 0.39 for the main effect of task switch, qualified by the interaction of switch × condition, *F*(1,42) = 21.1, *p* < 0.001, $\eta_{p}^{2}$ = 0.33.

##### Total work time

**FIGURE 2:**
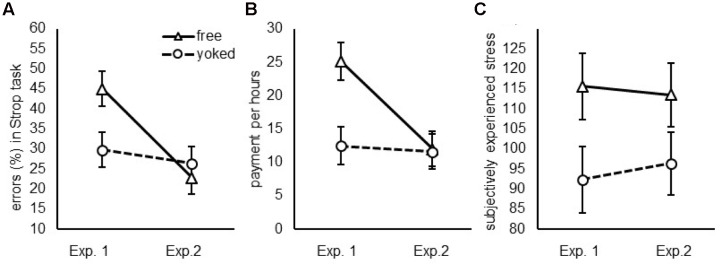
**Global measures of effort: (A)** The percentage of errors committed during the Stroop task, **(B)** the amount of payment participants considered as a fair compensation for performing the task and **(C)** the subjectively experienced stress during the experiment plotted for the free (triangles, straight line) and yoked (circles, dotted line) group for Experiment 1 (preview = 1000 ms) and Experiment 2 (preview = 200 ms). Error bars indicate the standard error of the mean.

When considering the sum of RT and choice time/time to start a trial (see **Figure 2**), total work time of participants in the free choice condition and in the cued condition did not differ significantly, *F*(1,42) = 3.1, *p* = 0.088, $\eta_{p}^{2}$ = 0.07. Participants responded faster in task switch than in task repetition trials, *F*(1,42) = 38.4, *p* < 0.001, $\eta_{p}^{2}$ = 0.48, and this switch advantage was larger in the free choice condition than in the cued condition, *F*(1,42) = 9.7, *p* = 0.003, $\eta_{p}^{2}$ = 0.19. Two-tailed one-sample *t*-tests against null revealed switch benefits both for participants in the free choice condition and in the cued condition, *t*(22) = 6.45, *p* > 0.001, *d* = 1.37 and *t*(21) = 2.36, *p* = 0.028, *d* = 0.51.

##### Task choices

Overall, Participants repeated tasks in 31.6% of the trials. This repetition rate does not differ significantly from the 25% repetition rate that would result if participants randomly chose task order, *t*(21) = 1.4, *p* = 0.168, *d* = 0.29. To further analyze task choices, we considered only trials in a block as long as all four stacks for each task had items. For this subsample of trials, participants repeated tasks in 26.8% of the trials. This repetition rate does not differ significantly from the 25% repetition rate that would result if participants randomly chose task order, |*t*| < 1. When switching between tasks, participants switched to the similar task in 30.0% of the trials and to the two other dissimilar tasks in 43.2% of the trials. The switch rate within task categories was significantly above chance of 25%, *t*(21) = 2.54, *p* = 0.019, *d* = 0.54, while switches to the two dissimilar tasks occurred less frequently than expected by change of 50%, *t*(21) = 1.82, *p* = 0.083, *d* = 0.38.

### Discussion

The present experiment aimed to elaborate on an experimental setting that allows to identify conditions supporting multitasking. We introduced an experimental set-up that requires task switching but allows parallel processing of alternative task items to contrast self-organization and externally controlled task switching. In addition, to local performance measures, we also assessed global subjective and objective measures for effort. Results revealed a rather interesting data pattern. First, participants responded slower, made more errors, and total work time was larger for task repetition trials than task switch trials. Thus, in contrast to the usually observed task switch costs (see Kiesel et al., 2010 for a review), here reversed switch costs, that is, switch benefits emerged. This finding can easily be explained because the items for the non-chosen task remained on the screen. Because of this possibility to preview the items for task switches (see **Figure 1**), participants were able to work on these items while they received feedback for the just performed task. The feedback was given for 1000 ms and consequently participants had ample time to work on the alternative tasks’ items after responding.

Second, participants repeated tasks more often than expected by chance. This finding seem at odds with the observation that participants were able to respond faster in task switch than in task repetition trials. Yet, based on typical task switching experiments (for an overview see e.g., Kiesel et al., 2010; Vandierendonck et al., 2010), we know that task switching requires an effortful reconfiguration process and participants might avoid this process. We will come back later to this issue in the general discussion. Additionally, when participants switched tasks, they more often switched to the similar task category (from the addition to the subtraction task and vice versa or from the letter to the month distance task and vice versa) than expected by chance. We take this as a hint that participants choose tasks such that task switching was facilitated.

Finally, and most interestingly, participants in the free choice condition seemed to be more fatigued than participants in the cued condition and they subjectively experienced more stress. This finding is at odds with the observation that participants in the free choice condition responded faster in task switch trials than participants in the cued condition. Thus, the objective and subjective assessment of overall effort contradict the performance measures. Usually one would assume that faster responses occur for easier and thus less stressful conditions. Consequently, based on the performance data one might have predicted that participants in the free choice condition would be less fatigued and stressed than participants in the cued group. To account for these findings, we assume that participants in the free choice condition experienced more effort when considering global measures because the requirement to schedule tasks in order to respond as fast as possible (i.e., in order to optimize local performance) is demanding and thus induces stress and leads to fatigue. Yet, participants in the free choice condition were faster in task switch trials when considering local measures because their task choice enabled them to act more efficiently and thus to take more advantage from the possibility to preview the items in case of task switches.

Before we elaborate more on such a possible trade-off between local performance benefits and global effort costs, we have to consider an alternative explanation of Experiment 1. Task instruction for the free-choice group stated that participants should avoid pre-determined strategy like for example rotating task-order clockwise and without repeating the same task more than 2 or 3 times in a row. Arguably, this is a considerable additional task demand that might explain why participants in the free choice group were more fatigued after the experiment compared to the cued group without this demand. Indeed, previous research on voluntary task switching has shown that instructions to avoid specific choice patterns is cognitive demanding and impairs local performance (e.g., Mayr and Bell, 2006). Therefore, it is possible that global costs in terms of increased fatigue in the free choice group were due to difference in task instruction.

Furthermore, it remains unclear whether the long preview in combination with the possibility to select freely tasks resulted in beneficial task performance/more fatigue or whether free choice alone would have been sufficient to induce these effects. More precisely, it remains to be tested whether self-organization of task selection could is effortful (in terms of global costs) even without any local performance benefits. In order to test these assumptions we decided to run a second Experiment without reduced possibility to preview the non-chosen items.

## Experiment 2

In Experiment 2, we applied a similar experimental procedure than in Experiment 1, but we now reduced the time of the feedback after responding to the item of a task to 200 ms. We hypothesize that this massive reduction of the possibility to preview the non-chosen items before participants can choose a task, changes task choice behavior so that free choice of the task sequence does no longer support local performance. Thus, we predict that in Experiment 2 with reduced possibility to preview the items, local performance in the free choice condition and the cued task switch condition should not differ. In addition, we assume that if global costs (increased fatigue) result from local performance benefits (total work time), we do not expect any difference in global costs in Experiment 2. Consequently, we hypothesize that the global assessment of effort for participants in the free choice and cued task switching conditions does not differ. In contrast, if global costs result from task choice processes irrespective of a stimulus-strategy or if global costs result from the demanding task switch instruction, global assessment of effort should be increased for participants in the free choice compared to the cued task switching group.

### Method

#### Participants

Forty-eight participants (eight men, three left-handed, 18–56 years) took part in exchange for course credits or 10 were analyzed. All participants were tested in sessions that lasted approximately 90 min. The first ten participants were assigned to the voluntary group and the next ten participants were yoked to the first ten participants and tested under the cue condition. This procedure was repeated for the remaining participants.

#### Stimuli and Procedure

Stimuli and procedure was identical to Experiment 1 except for the following. In the main Experiment, we reduced the time of the feedback to 200 ms. When a response was registered, this response was shown in the middle of the screen in a square with green background in case of correct responses or red background in case of an error. During this feedback, the items for all four tasks remained on the screen. After 200 ms, the feedback disappeared and a new item appeared at the location of the just chosen task. For the subjective measures, we did not assess the IPANAT in Experiment 2 because this measure was not sensitive in Experiment 1.

### Results

#### Global Measures to Assess Fatigue/Stress

##### Stroop task

The first trial of the block was excluded and for the RT analyses, only correct trials were included. Participants responded slower, *F*(1,46) = 23.14, *p* < 0.001, $\eta_{p}^{2}$ = 0.335 and made more errors, *F*(1,46) = 26.24, *p* < 0.001, $\eta_{p}^{2}$ = 0.363 in Stroop incongruent compared to Stroop congruent trials. Performance of participants in the free choice condition and in the cued condition did not differ, *F* < 1 for RT and errors see **Figure 2** and **Table 1** for means.

##### Subjectively experienced stress

Neither participants’ reported stress did not differ between the conditions, *t*(46) = 1.45, *p* = 0.15, nor the amount of payment per hours that participants demanded for compensation for a future participation did differ between conditions, |*t| <* < 1.

##### Affect

As in Experiment 1, mood ratings did not differ between groups, *F* < 1.

To summarize, participants in the free choice condition and participants in the cued condition did not differ significantly regarding fatigue and experienced stress.

#### Local Task Switching Performance

The first trial in each block was not analyzed. Post-error trials (7.3%) and RTs that exceeded more than 2.5 SDs from the cell mean for each condition (4.6%) were removed from all analyses. Additionally trials with erroneous responses (5.5%) were removed from all analyses (except analysis of error data). As in Experiment 1, a repeated-measures ANOVA with the factors *condition* (free choice, cued) and *task transition* (repeat, switch) was used to analyze the data.

##### Task performance (reaction times and errors)

Participants in the free choice condition responded faster than participants in the cued condition, *F*(1,46) = 15.6, *p* < 0.001, $\eta_{p}^{2}$ = 0.25. The response times did not differ significantly in task switch and in task repetition trials, *F*(1,46) = 2.65, *p* = 0.11, $\eta_{p}^{2}$ = 0.055, and there was no significant interaction of switch × condition, *F*(1,46) = 0.5, *p* = 0.82, $\eta_{p}^{2}$ = 0.001. Analysis of error rates did not reveal a difference for participants in the free and yoked group, *F*(1,46) = 0.3, *p* = 0.86, $\eta_{p}^{2}$ = 0.001. Further, there was no significant difference between switch and repetition trials, *F*(1,46) = 2.11, *p* = 0.15, $\eta_{p}^{2}$ = 0.044, and no significant interaction, *F*(1,46) = 0.14, *p* = 0.71, $\eta_{p}^{2}$ = 0.003.

##### Task choice times

Regarding the time to choose a task/start the trial, participants in the free choice condition took longer than participants in the cued task condition, *F*(1,46) = 19.2, *p* < 0.001, $\eta_{p}^{2}$ = 0.29. Yet, task choice times did not differ significantly for task switches or repetitions, *F*(1,46) = 1.07, *p* = 0.31, $\eta_{p}^{2}$ = 0.023 for the main effect of task switch, and for the interaction of switch x condition, *F*(1,46) = 1.12, *p* = 0.30, $\eta_{p}^{2}$ = 0.024.

##### Total work time

When considering the sum of RT and choice time/time to start a trial (see **Figure 2**), response times did not differ for participants in the free choice condition and in the cued condition, *F*(1,46) = 1.49 *p* = 0.23, $\eta_{p}^{2}$ = 0.031. Further, total work time did not differ for task switch and task repetition trials, *F*(1,46) = 0.046, *p* = 0.83, $\eta_{p}^{2}$ = 0.001, and the interaction of switch × condition was not significant, *F*(1,46) = 0.748, *p* = 0.39, $\eta_{p}^{2}$ = 0.016.

##### Task choices

Overall, Participants repeated tasks in 50.9% of the trials. This repetition rate is significantly larger than the 25% repetition rate that would result if participants randomly chose task order, *t*(23) = 4.32, *p* < 0.001, *d* = 0.88. To further analyze task choices, we considered only trials in a block as long as all four stacks for each task had items. For this subsample of trials, participants repeated tasks in 48.5% of the trials. This repetition rate is significantly larger than the 25% repetition rate that would result if participants randomly chose task order, *t*(23) = 3.88, *p* < 0.001, *d* = 0.79. When switching between task, participants switched to the similar task in 22.8% of the trials and to the two other dissimilar tasks in 28.6% of the trials. The switch rate within task categories did not significantly differ chance of 25%, |*t*| < 1 while switches to the two dissimilar tasks occurred less frequently than expected by change of 50%, *t*(23) = 5.84, *p* < 0.001, *d* = 1.19.

## Between Experimental Comparison

To compare the results of Experiments 1 and 2, we added the between-factor *Experiment* to the respective ANOVAs reported for Experiments 1 and 2. Only effects of interest, i.e., the interaction with *Experiment* are reported.

### Global Measures to Assess Fatigue/Stress

For the error rates in Stroop task, the difference in the free compared to the cued condition was more pronounced in Experiment 1 (Δ = 15.23%) than in Experiment 2 (Δ = -3.51%), as indicated by the significant interaction between Experiment and group (free, cued) and *F*(1,88) = 4.80, *p* = 0.031, $\eta_{p}^{2}$ = 0.052. While the difference for the free and cued switching group was stronger for subjectively reported stress in Experiment 1 (Δ = 23.31) compared to Experiment 2 (Δ = 17.12), this difference was not significant, *F* < 1. However, the difference between free and cued switching group in terms of the amount of payment per hours that participants demanded for compensation was significant (Experiment 1, Δ = 12.67 ; Experiment 2, Δ = 0.43 €), *F*(1,88) = 5.07, *p* = .027, $\eta_{p}^{2}$ = 0.056.

### Local Task Switching Performance

#### Task Performance (Reaction Times and Errors)

The difference in task performance for free and yoked groups was not different between Experiments (four-way interaction with *F* < 1). Although there was a tendency for overall switch benefits in Experiments 1 (Δ = 425 ms) and switch costs Experiment 2 (Δ = 56 ms) irrespective of free/ yoked group, this difference was only marginal significant, *F*(1,89) = 3.14, *p* = 0.08, $\eta_{p}^{2}$ = 0.034. For errors rates, the four-way interaction was marginal significant, *F*(1,89) = 3.14, *p* = 0.08, $\eta_{p}^{2}$ = 0.034, showing a tendency for greater switch benefits in Experiments 1 for the free compared to yoked group (Δ = 2.71 %) compared to Experiment 2 with the reverse patter, namely a switch benefit for the yoked group (Δ = 0.47%).

#### Task Choice Times

The difference in switch costs/benefits for free compared to yoked groups was significantly stronger in Experiment 1 compared to Experiment 2, *F*(1,89) = 5.24, *p* = 0.024, $\eta_{p}^{2}$ = 0.056. As indicated by the individual analysis, participants in Experiment 1 showed a pronounced switch *benefit* in the free choice group against the yoked group (Δ = 334 ms), while participants in Experiment 2 showed strong switch *costs* in the free choice group against the yoked group (Δ = 251 ms).

#### Total Work Time

**FIGURE 3:**
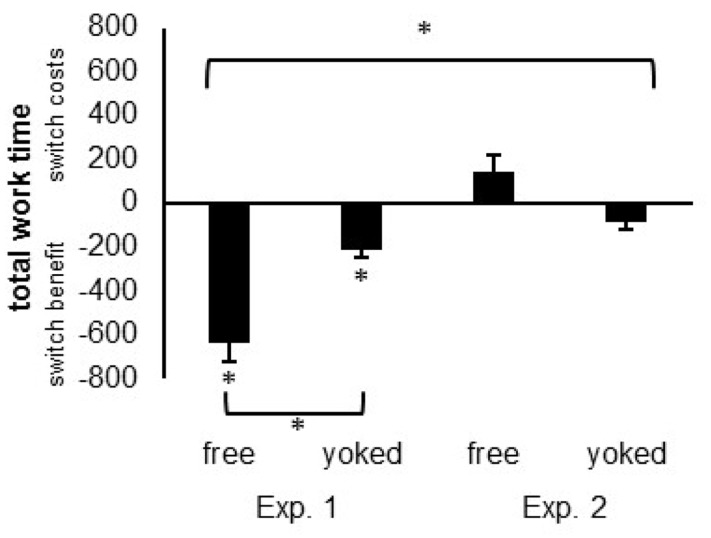
**Local measures of performance costs displayed as the switch costs/benefits (repetition time – switch time) calculated for the total work time (task choice + task performance) for the free and cued group of Experiment 1 (preview = 1000 ms) and Experiment 2 (preview = 200 ms).** Asterisks indicate significant differences. Error bars indicate the standard error of the mean.

The difference in switch costs/benefits for free compared to yoked groups was significantly stronger in Experiment 1 compared to Experiment 2, *F*(1,89) = 4.79, *p* = 0.031, $\eta_{p}^{2}$ = 0.051. As indicated by the individual analysis, participants in Experiment 1 showed a pronounced switch *benefit* in the free choice group against the yoked group (Δ = 425 ms), while participants in Experiment 2 showed strong switch *costs* in the free choice group against the yoked group (Δ = 56 ms) (see **Figure 3**).

#### Task Choices

Repetition rate was higher for Experiment 2 compared to Experiment1, *t*(44) = 2.75, *p* = 0.009, *d* = 0.082. Furthermore, within switches and across task switches were lower in Experiment 2 compared Experiment 1 |*t*| (44) = 2.14, *p* = 0.038, *d* = 0.063 and |*t*| (44) = 2.77, *p* = 0.08, *d* = 0.082.

### Discussion

In Experiment 2, we assessed local performance measures and global measures of stress and fatigue in a setting that resembles the setting of Experiment 1. Yet, in contrast to Experiment 1, the feedback screen after responding in a trial was presented for 200 ms only, before the new item for the just chosen task occurred (while the items for the non-chosen tasks remained the same). This reduction of preview for the non-chosen items lead to a rather different response pattern than in Experiment 1. Local performance measures showed no advantage for switch compared to repetition trials. Arguably, a 200 ms preview is not sufficient to facilitate task switching significantly. Additionally, participants in the free choice condition responded faster, yet they took longer to choose a task than participants in the cues condition. When considering the combined measure of total work time, there was no significant difference for both conditions. It seems that participants in the free choice group waited to indicate their task choice such that they were able to respond faster. Yet, overall participants’ choice behavior could not optimize task performance due to the lack of preview. Similarly, global measures to assess fatigue and stress did not differ in the free choice and the cued task switching group. Thus, taken together there were no significant local differences and no global differences (only in one measure marginally different) in the free choice and the cued group in Experiment 2. This suggests that preview is actually necessary to choose tasks in a way that supports optimized behavior.

Please note that this finding is also suitable to rule out two objections against Experiment 1. First, one might suppose that participants in the free choice condition experienced more effort than participants in the cued condition, because they were instructed to choose a task without following a fixed task sequences and without repeating tasks too often. Yet, instructions how to choose tasks were the same in Experiments 1 and 2. Thus, task choice instructions itself cannot explain differences between free choice and cued group.

Further, task choice behavior in Experiment 2 revealed that participants repeated tasks more often than expected by chance. Due to the decreased preview, the advantages to switch tasks were reduced and thus participants preferred the less demanding task repetition option. When participants switched trials, they did not switch more often than expected by chance to the similar task category. It thus seems that the reduced possibility to preview likewise reduced the possibility to optimize task scheduling.

## General Discussion

In the present study, we assessed local performance measures as well global measures for effort in a multitasking setting comparing free choice of task order and cued task order. Participants in the free choice condition scheduled task order when switching between four different tasks. After performing an item for one task, participants received feedback either for 1000 ms (Experiment 1) or for 200 ms (Experiment 2) while the items for the non-chosen tasks remained the same. Consequently, participants could use the feedback time as a preview to prepare for the alternative tasks and in the free choice condition to choose a task order that optimizes performance. Results in Experiment 1 indicated that participants in the free choice condition were faster than participants in the cued condition, yet global measure of effort revealed that they were more stressed and fatigue after the experiment. In contrast, in Experiment 2 with largely reduced preview, neither local nor global measures for the free choice and the cued group differed.

To account for these results, we speculate that there are three mechanisms that interact with each other:(i) advance item processing due to preview, (ii) reconfiguration required in task switch trials, and (iii) a task choice process (in free choice condition) that aims to optimize reconfiguration and task processing.

First, we suppose that participants responded faster in task switch than in task repetition trials, because the preview time allowed participants to prepare (and possibly even solve) the next task before the start of a trial.

**FIGURE 4:**
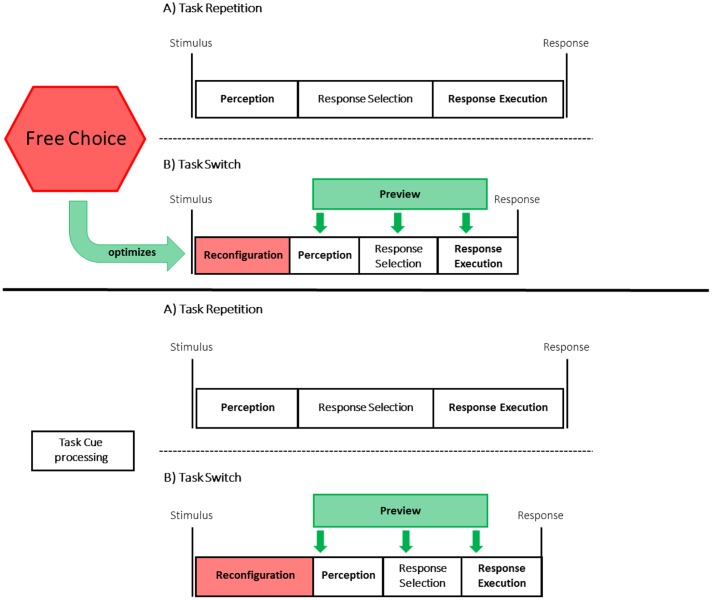
**In both the free choice (upper) and the cued condition (lower) preview facilitates responding in task switch compared to task repetition trials.** Yet, task switching requires reconfiguration; thus, the advantages of preview are attenuated. In the free choice condition **(upper)**, the process to freely choose a task is effortful and time-consuming, yet it aims to optimize reconfiguration (and possible) task processing and thus facilitates responding in switch trials in the free choice condition.

Second, we assume that in addition to the possibility to work in advance on the task-switch items, another process impacts on task choice/task performance that prevents frequent task switching. Task switching requires a reconfiguration process to adopt the new task set (see **Figure 4**). This reconfiguration process is an executive control process and requires cognitive resources (e.g., Rogers and Monsell, 1995; Meiran, 1996; Koch, 2001; Hoffmann et al., 2003; Plessow et al., 2011; Plessow et al., 2012). To avoid the cognitive demand that is related to task switches, participants prefer to repeat a task. Thus, our results seem to be in line with the “law of least mental effort” (Kool et al., 2010, p. 678).

Indeed, research in the voluntary task switching paradigm further supports this assumption that participants avoid cognitive demand. A number of studies revealed that when participants were instructed to randomly choose a task, they usually repeated tasks more often than expected by chance (e.g., Arrington and Logan, 2004, 2005; Mayr and Bell, 2006; Yeung, 2010; Reuss et al., 2011). This repetition bias seems reasonable because in these voluntary task switching experiments, task switch cost emerged. Yet, in the current setting participants responded faster in task switch trials because the items for the tasks remained on the screen and because of this preview possibility participants were able to work on the previously non-chosen items (the items that would be chosen in task switch trials) while the feedback screen was presented. Nevertheless, despite responding faster in task switch trials, participants did not prefer task switches over task repetitions. This observation is interesting because it might question the assumption that self-organization, that is, free choice of task order is suitable to optimize overall task performance. Further research is needed to clarify whether participants are able to balance performance benefits (or costs) in an experimental setting with the effort of task reconfiguration processes. Currently, we can only speculate why participants did not choose the faster option more often. It might be that participants were not aware that they would be faster in task switch trials or alternatively, the reconfiguration process might induce some level of conflict and thereby negative affect (e.g., Botvinick, 2007; Dignath et al., 2015).

Third, we assume that the task choice process for participants in the free choice group is not only affected by the necessity to reconfigure but also itself impacts on reconfiguration and task performance. Here, a central conjecture is that the task choice process aims to optimize task performance and effort related to task processing (e.g., Shenhav et al., 2013). In the setting of the present study, the task choice process has to balance the tendency to (1) avoid task switches to avoid reconfiguration processes, and (2) to exploit the preview possibility and thus to prefer task switches. Interestingly, results revealed that participants responded faster in task switch than in task repetitions trials, yet they did not switch tasks more often (indeed descriptively they even repeated tasks more often) than would be expected for random task choices. Thus, the usual conclusion that fast RTs indicate easy task conditions that are preferred by participants does not hold in this setting.

In addition, the task choice process is an executive control process and as such requires cognitive resources. Despite that participants in the free choice condition needed less time than participants in the cued condition to perform a task especially in task switch trials, subjective evaluation measures and after-effects in a Stroop task indicated that the free choice condition is more stressful/effortful than the cued task condition. Based on this observation, we conclude that assessment of overall effort (with subjective and objective measures) is an additional factor that should be considered in addition to performance data when comparing different multitasking conditions.

Taken together, participants in the free choice condition were more fatigued than participants in the cued condition and they subjectively experienced more stress. Thus, despite participants in the free choice condition needed less time than participants in the cued condition to perform a task especially in task switch trials, subjective evaluation measures and after-effects in a Stroop task indicated that the free choice condition is more stressful/effortful than the cued task condition. To conclude the present experiment suggests that task organization in multitasking depicts a trade-off. While self-organization of task scheduling can optimize task performance during multitasking, it comes at the costs of more fatigue after multitasking.

## Ethics Statement

Funding for this research was granted by the Deutsche Forschungsgemeinschaft without the necessity of an approval by an ethics committee. The study was conducted with healthy participants who gave informed consent regarding data collection. All procedures performed in studies involving human participants were in accordance with the ethical standards of the institutional and national research committee and with the 1964 Helsinki declaration and its later amendments or comparable ethical standards. Informed consent was obtained from all individual participants included in the study.

## Author Contributions

AK and DD designed the study. AK analyzed the data and wrote major parts of the manuscript. DD discussed the analysis and contributed to write the manuscript.

## Conflict of Interest Statement

The authors declare that the research was conducted in the absence of any commercial or financial relationships that could be construed as a potential conflict of interest.

## Footnotes

1 Indeed, repeating non-chosen items allows for very different optimization processes compared to situations with new items for each task. In the latter case (and with the requirement to choose only one task) it would be best to choose the easiest task that can be performed fastest. In most trials, this would mean that participants repeat the task without any further consideration of task sequences.

2 Participants also answered to an adaptation of the “NASA Task Load Index” (NASA-TLX, Hart and Staveland, 1988), yet due to failures in the translation these data could not be analyzed.

3 The overall error rate (*M* = 38.1%) in the Stroop task was considerably high. However, this is a typical pattern for a brief manual Stroop task that requires participants to memorize a 4-color-key mapping rule without training and a considerable short response deadline (c.f. Dignath and Eder, 2015).

4 Degree of freedoms differs for this analysis because some participants did not answer this question.

5 Data of one participant who did not fill out the IPANAT is missing.
